# Supplementary material for: Tramesan Elicits Durum Wheat Defense against the Septoria Disease Complex
Source: Biomolecules. 2020 Apr 14;10(4):608. doi: 10.3390/biom10040608 (PMC7225966; doi:10.3390/biom10040608)

Additional images of Svevo cultivar plots inoculated with *Zymoseptoria tritici* in field

File 083: Plot 1054 **Svevo T**
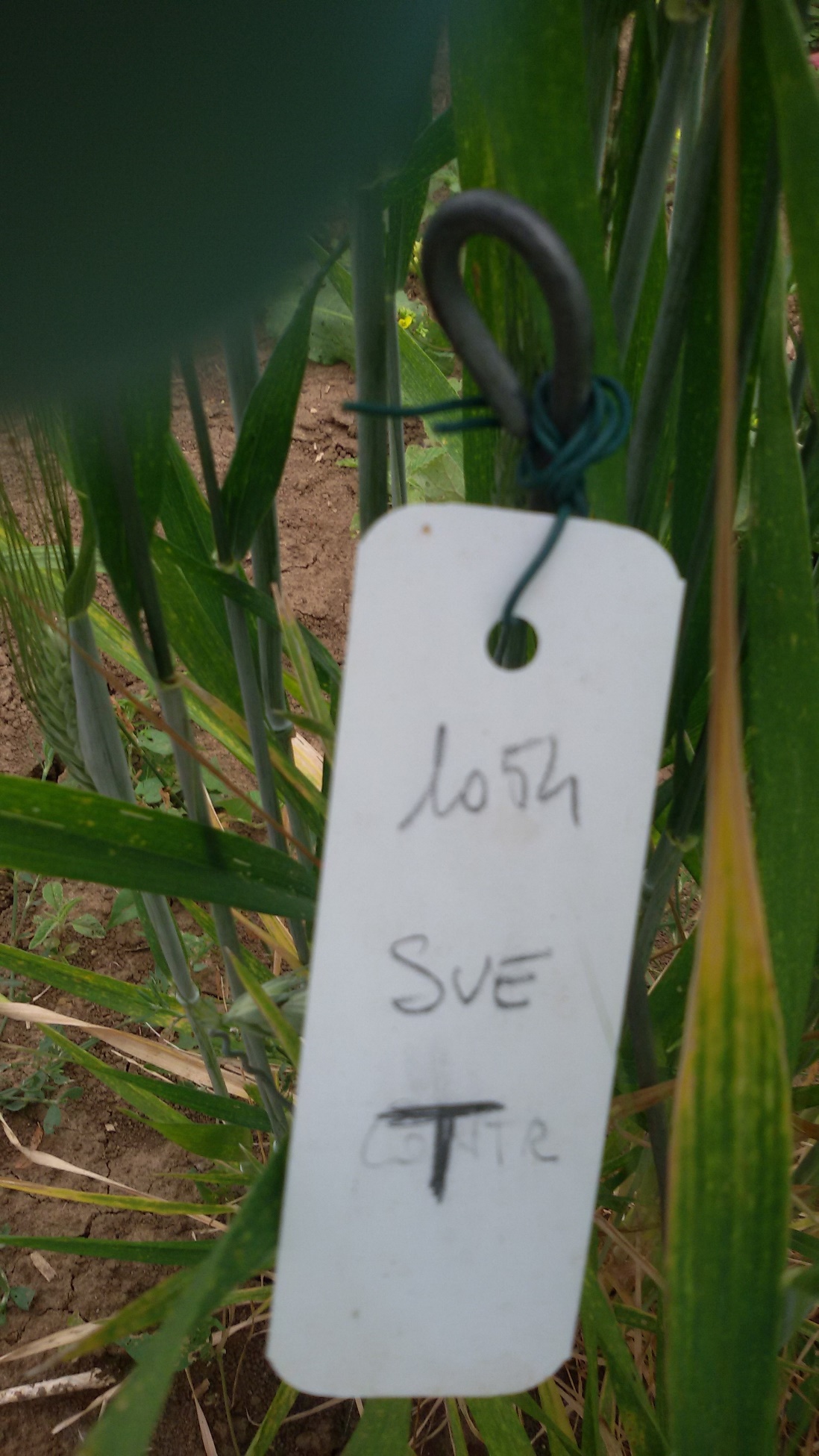


File 085: Plot 1054 **Svevo T**
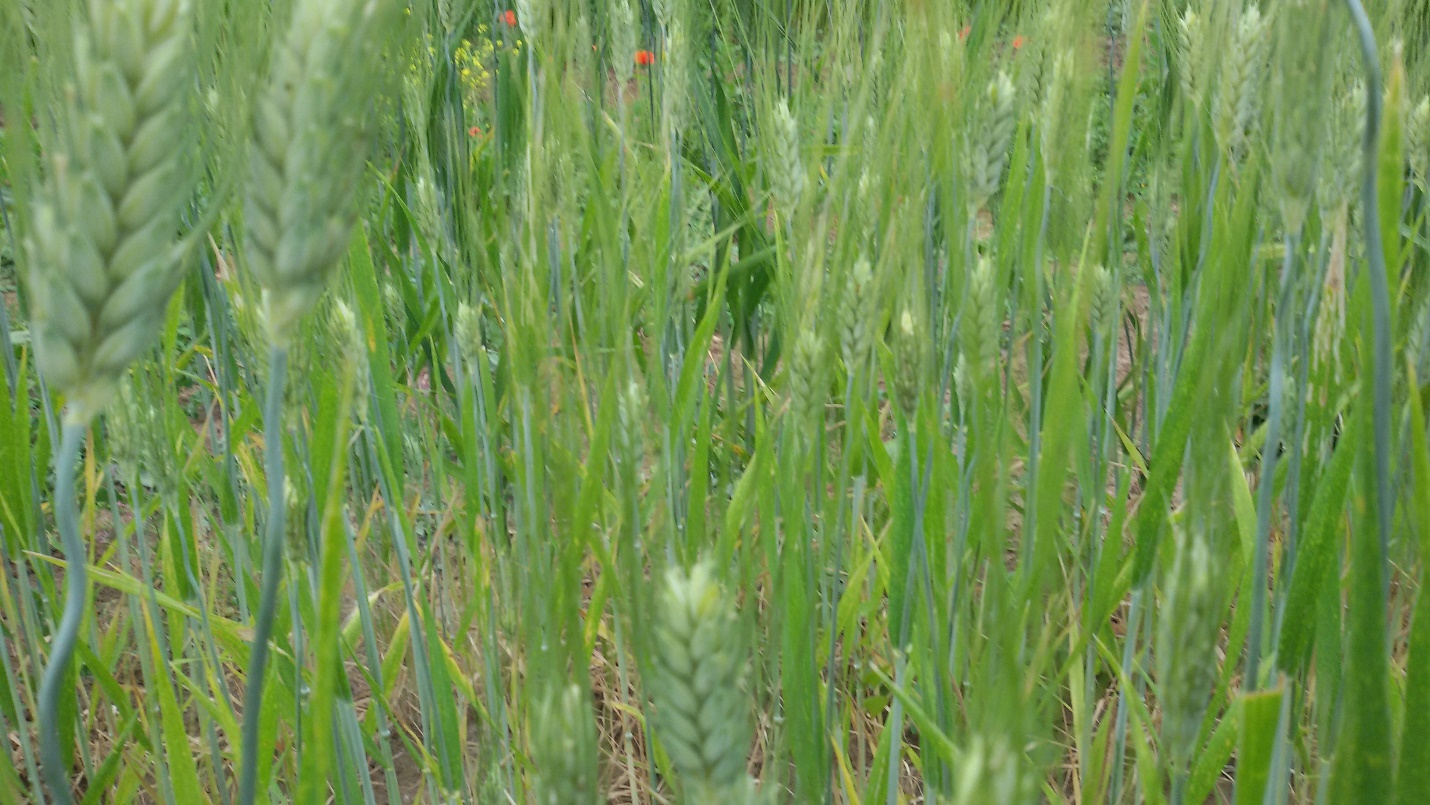


File 090: Plot 1051 **Svevo INF**


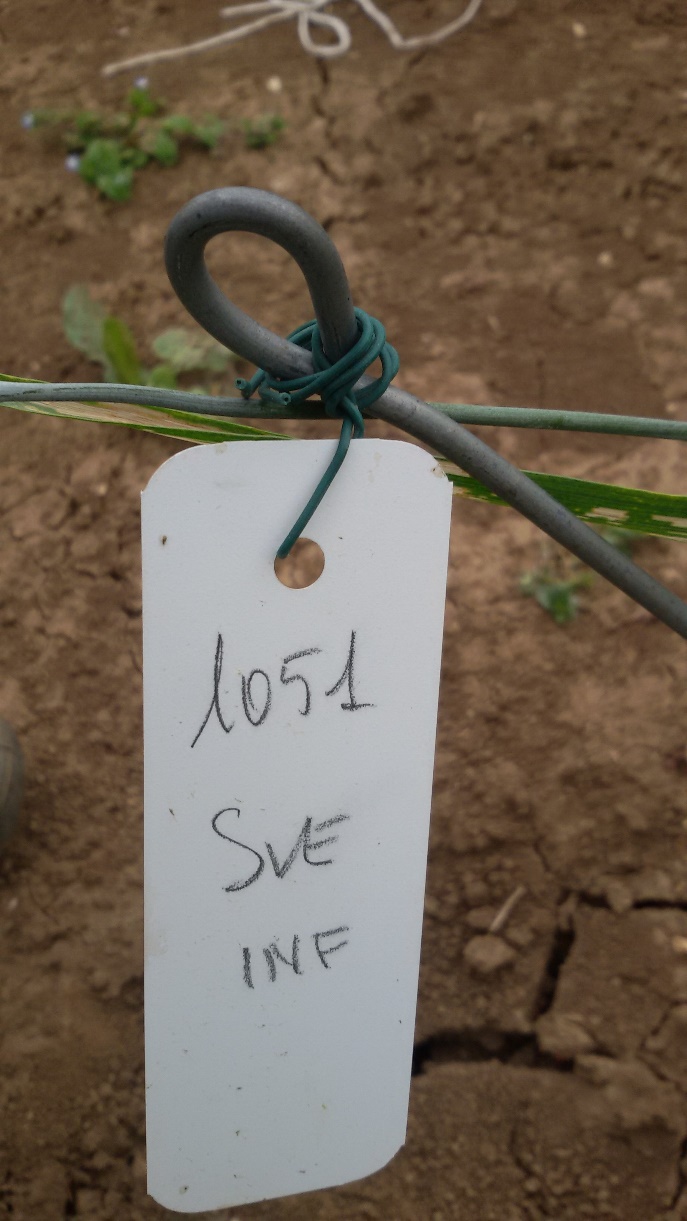


File 116: Plot 1051 **Svevo INF**
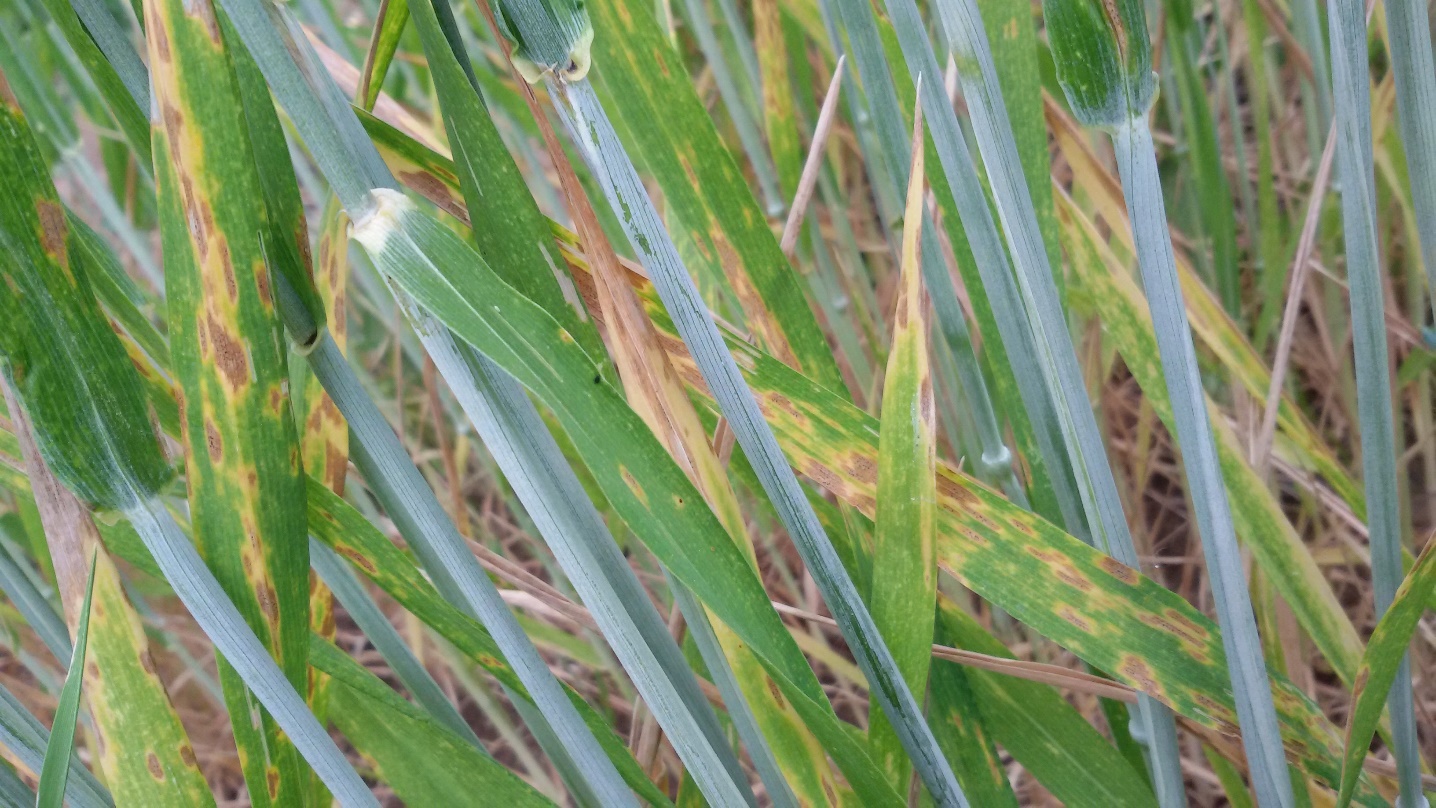


File 134: Plot 1058 **Svevo T + INF**


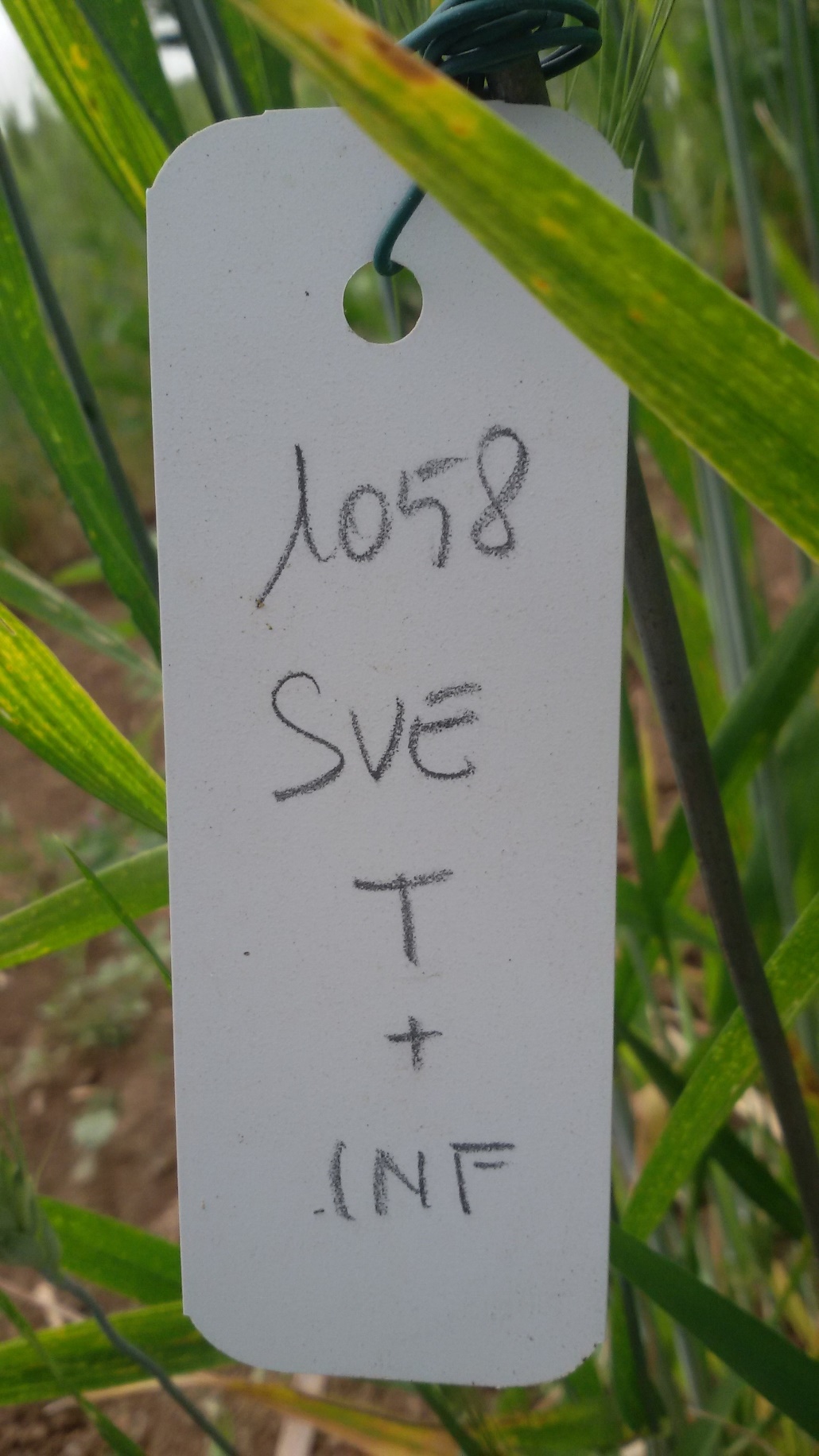


File 136: Plot 1058 **Svevo T + INF**


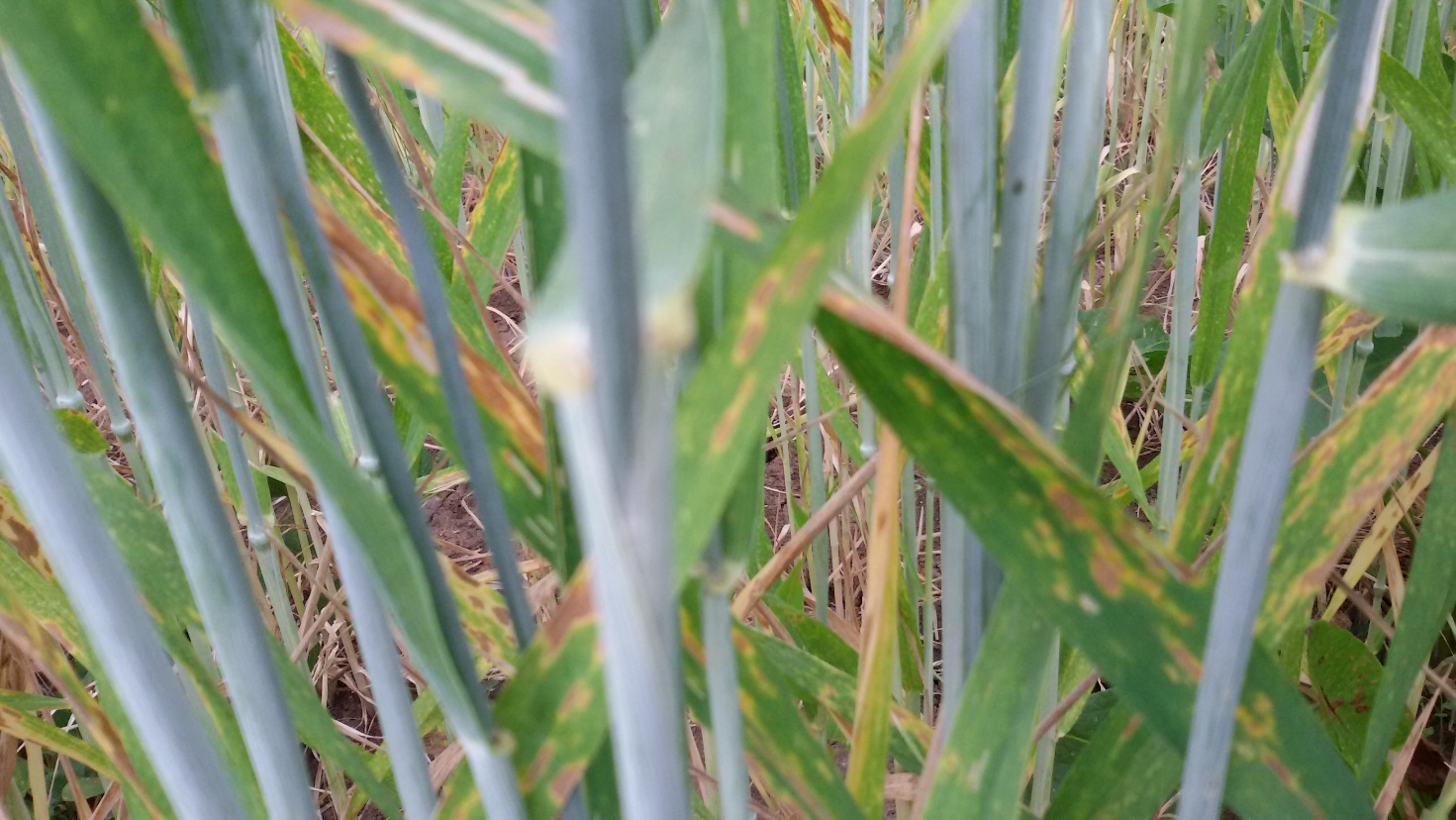

Supplement: Supplementary file 1 [file biomolecules-10-00608-s001.zip › supplementaries/Supplementary Figure 1.docx]
