# Supplementary material for: Tramesan Elicits Durum Wheat Defense against the Septoria Disease Complex
Source: Biomolecules. 2020 Apr 14;10(4):608. doi: 10.3390/biom10040608 (PMC7225966; doi:10.3390/biom10040608)
Supplement: Supplementary file 1 [file biomolecules-10-00608-s001.zip › supplementaries/Supplementary Table 1.docx]

**Supplementary Table 1**. List of Primers used in this study

| **Target gene** | **Sequence (5’-3’) For** | **Sequence (5’-3’) Rev.** |
| --- | --- | --- |
| PR1 | CATGCACCTTCGTATGCCTAACT | TGGCTAATTACGGCATTCCTTT |
| Chit | GGGTGGACCTGCTGAACAAT | AGAACCATATCGCCGTCTTGA |
| PAL | GTCGATTGAGCGTGAGATCAAC | CACGGGAGACGTCGATGAG |
| PER | TGCTTTGTCCAAGGCTGTGA | GACCCGCGTTTTGTTCCA |
| NADPHox | CCATGTTCGGCAACTTGGTG | AAGGGCCATCGATCAGAAGC |
| PR4 | ATCATTACTACCGGCCAGCG | GTCCACCCGTACTTGGACC |
| MCa2 | ACAGTGGTGGCCTACTGGAC | AGTGAGACCATCCCCAGTTG |
| Cerk1 | TTCCCATTGGCTCGTTTCG | TCACATGAACGTGGCGCTAA |
| MPK3 | TACATGAGGCACCTGCCGCAGT | GGTTCAACTCCAGGGCTTCGTTG |
| PnToxA | GGAAAATACTCTCACTCGGGGG | AGGCAAAAGCTTCCTCTACCT |
| PnTox1 | CTCACGTTTGAGGGCTTAGG | GGATGCAATAGAGCAGCAGA |
| PnTox3 | CAACGCTGCTTCAACGACAA | ACTCTTCCTGCTGGGCATTC |
